# **THE ROLE OF CAPSULE ENDOSCOPY IN URGENT EVALUATION OF OBSCURE GASTROINTESTINAL BLEEDING: A CASE SERIES OF MECKEL DIVERTICULUM** *.*

**DOI:** 10.1590/0102-672020180001e1409

**Published:** 2018-12-06

**Authors:** Marco SILVA, Hélder CARDOSO, Armando PEIXOTO, Susana LOPES, Ana Luísa SANTOS, Sara GOMES, Guilherme MACEDO

**Affiliations:** 1 Centro Hospitalar de São João, Gastroenterologia, Porto, Portugal

**Keywords:** Meckel diverticulum, Endoscopy, gastrointestinal, Endoscopic capsule, Endocapsule, Gastrointestinal hemorrhage, Divertículo ileal, Cápsula endoscópica, Endocápsula, Endoscopia gastrointestinal, Hemorragia gastrointestinal

## INTRODUCTION

Meckel’s diverticulum (MD) is the most common congenital malformation of the gastrointestinal tract^4^. In adults, it is usually clinically silent, but can be found incidentally, or may present with a variety of clinical manifestations including gastrointestinal bleeding, intussusception, intestinal obstruction or perforation^3^ ^,^ ^4^. By other hand, videocapsule endoscopy (VCE) is a powerful diagnostic tool that is especially useful in imaging the small intestine and management of patients with obscure gastrointestinal bleeding^5^. 

The authors conducted a retrospective analysis of patients with MD diagnosed by VCE, between 2006 and 2015, in a tertiary referral center. All cases were followed for at least 18 months after the diagnosis. 

## CASES REPORT

### CASE 1

A 15-year-old caucasian male, with non-relevant past medical history. He had no prior history of change in bowel habits or gastrointestinal bleeding and was admitted to the emergency department after an episode of lipothymy. He complained of asthenia and hematochezia since the day before. The laboratory tests showed hemoglobin of 10.8 g/dl. The upper endoscopy was normal and the ileocolonoscpy showed ileal nodular hyperplasia with blood and clots in the ileum. Twenty-four hours later the hemoglobin dropped to 7.9 g/dl and on physical examination he was pale, diaphoretic and hypotensive. VCE (Endocapsule Olympus^®^) performed 24 h after admission identified the bleeding source as an active bleeding (oozing) from a small diverticulum like orifice in the middle ileum. 

### CASE 2

**FIGURE 1:**
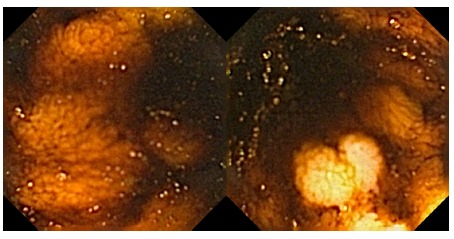
Case 2 videocapsule endoscopy imaging

A 16-year-old caucasian female had past medical history irrelevant. She was admitted due to melena lasting for 24 h. On admission she initiated hematochezia and pale but normotensive. The hemoglobin was 12.9 g/dl on admission but dropped to 7.1 g/dl 24 h later, requiring blood transfusions. Also the upper endoscopy was normal and on the ileocolonoscpy she had fresh clots in the ileum, without other relevant lesions. Then a VCE (PillCam SB 2^®^) was used revealing a luminal duplication on the terminal ileum (Figure 1). 

### CASE 3

A 14-year-old caucasian male was admitted due to melena. He had melena six months previously, but endoscopy and ilecolonoscopy performed at that time did not showed lesions with active bleeding. On admission, he was pale and hypotensive and laboratory workup revealed hemoglobin of 7.0 g/dl. After hemodynamic resuscitation, an upper endoscopy was performed but, also, did not showed any relevant alterations. The abdominal computer tomography (CT) and the Meckel scan with 99mTc-Na-pertechnetate also did not show relevant findings. At this time a VCE (PillCam SB 2^®^) was performed and luminal duplication in the terminal ileum, with signs of active bleeding, was visualized (Figure 2). 

**FIGURE:**
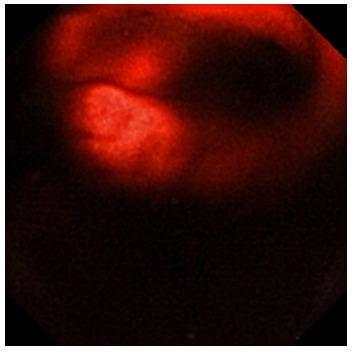
2 - Case 3 videocapsule endoscopy imaging

### CASE 4

A 17-year-old caucasian male was admitted in hypovolemic shock and rectal bleeding. He had hemoglobin of 4.9 g/dl on admission. After blood transfusions and hemodynamic resuscitation and upper endoscopy followed by ileocolonoscopy were performed, which did not showed lesions with active bleeding. The CT scan also did not showed any relevant lesions. VCE (PillCam SB 2^®^) was performed and identified a diverticular ulcerated lesion on the middle ileum with active bleeding. 

### CASE 5

A 19-year-old caucasian man was admitted with melena, that also have occurred two days previously. In that time, he had hemoglobin of 13.9 g/dl and was discharged with indication for symptoms surveillance. In admission laboratory workup performed revealed hemoglobin of 7.3 g/dl. After two blood transfusions, upper endoscopy and colonoscopy were performed without identification of any bleeding lesions. Abdominal-CT scan also did not showed anything relevant. Meckel scan with 99mTc-Na-pertechnetate was negative for heterotopic gastric tissue in the small bowel area. Due to hemodynamic instability an angiography was performed, also without identifying the source of bleeding. VCE (PillCam SB 3^®^) revealed a luminal duplication consistent with MD, but without active bleeding

All five patients were submitted to surgical excision of the MD and the histological examination confirmed the diagnosis. All of them presented good outcome without bleeding recurrence

## DISCUSSION

MD is a remnant of the omphalomesenteric duct, typically located at the antimesenteric site of the ileum, approximately 40-100 cm proximal to the ileocecal valve^7^. Although it affects 2-4% of the general population, symptomatic cases are just 4-16%^8^. Over 60% of patients are up to two years old and it is rarely seen in older children or adults^8^. Although, owing to improved endoscopic techniques for the small bowel such as VCE and balloon enteroscopy, it is increasingly recognized as a potential bleeding site in adults. The most common complication of MD is gastrointestinal bleeding^8^. In children it is a well-known cause of acute, painless intestinal bleeding^8^.

Despite the availability of modern imaging techniques diagnosis is challenging^8^. Upper endoscopy and colonoscopy are the investigation techniques of choice to look for the cause of gastrointestinal bleeding^8^. In all these patients upper and lower endoscopy failed to detect the bleeding site. Arteriography is not always diagnostic because it can only detect bleeding of at least 1-2 ml/min^8^. One of the newer technologies that expand the diagnostic capabilities in the small intestine is VCE. Some cases of diagnosis made with it have been reported^6^. Endoscopic appearance is characterized by two lumina, a thickened bridge, ulcer, and occasionally direct visualization of ectopic gastric mucosa^1^. However, in case of active bleeding, the diverticulum itself may be missed^2^.
